# Childhood Idiopathic Epilepsy in Saudi Arabia From 1990-2019: Analysis of the Global Burden of Disease Data, 2019

**DOI:** 10.7759/cureus.74549

**Published:** 2024-11-27

**Authors:** Kamel A Alenazi, Ahmad A Alanezi

**Affiliations:** 1 Department of Pediatrics, College of Medicine, Imam Mohammad Ibn Saud Islamic University, Riyadh, SAU; 2 Department of Pediatrics, King Saud University Medical City, Riyadh, SAU

**Keywords:** children, epidemiology, global burden of disease, idiopathic epilepsy, saudi arabia

## Abstract

Introduction

In Saudi Arabia, the epidemiological estimates of childhood epilepsy are lacking. However, only a handful of studies have been performed to explore the epidemiology of childhood epilepsy. The objective of this study was to identify the burden of childhood idiopathic epilepsy in Saudi Arabia from 1990 to 2019 using Global Burden of Disease (GBD) 2019 study data.

Methods

Data on the burden of childhood idiopathic epilepsy (children aged 0-14 years) in Saudi Arabia from the GBD 2019 study was reviewed and presented. The outcome measures of the study were incidence, prevalence, deaths, years of life lost (YLLs), years lived with disability (YLDs), and disability-adjusted life-years (DALYs) of childhood idiopathic epilepsy.

Results

In Saudi Arabia, the incident counts, prevalence, deaths, YLLs, YLDs, and DALYs were 5,365, 26,275, 58, 4,808, 9,466, and 14,273, respectively in 1990. The incidence [25.32% (95% uncertainty interval (UI): -64.00 to 379.03)], prevalence [35.40% (95% UI: -61.37 to 419.98)], and YLDs [7.99% (95% UI: -74.80 to 371.75)] rate (per 100,000 population) increased between 1990 and 2019. On the other hand, the death [-69.21% (95% UI: -82.54 to -34.86)], YLLs [-69.97% (95% UI: -83.13 to -35.81)], and DALYs [-18.27% (95% UI: -73.15 to 119.87)] rate (per 100,000 population) dropped between the same duration.

Conclusion

In the last three decades, the incidence and prevalence of childhood idiopathic epilepsy have modestly increased in Saudi Arabia, but the number of deaths has fallen substantially. Further research studies are needed from Saudi Arabia to understand the regional, gender-based, and socioeconomic differences of childhood idiopathic epilepsy and its impact on the psycho-cognitive development and quality of life of children.

## Introduction

Idiopathic epilepsy is a subtype of generalized epilepsy of genetic origin or without an identifiable morphological, infectious, metabolic, or immune system-related trigger [1]. According to the Global Burden of Disease (GBD) Collaboration Report 2017, idiopathic epilepsy was responsible for approximately 5% of the total neurological disability-adjusted life years (DALYs) and 1.3% of all the mortalities between 1990 and 2015. Idiopathic epilepsy was listed as the fifth-most frequent neurological disorder, preceded by stroke, migraine, dementia, and meningitis [2].

Up-to-date and improved estimations of children with idiopathic epilepsy are vital to measuring the burden of disease and the funds and resources necessary to address the needs of these children as directed by the United Nations (UN) in Goal 3 ‘Good Health and Well-Being’ of sustainable development goals (SDGs) [3, 4]. Recent estimates of the GBD 2019 report suggest that idiopathic epilepsy was responsible for 13.1 million (95% uncertainty interval [UI]: 9.99 to 16.7) global disability-adjusted life years (DALYs) in 2019. To our knowledge, in the Kingdom of Saudi Arabia, the epidemiological estimates of childhood epilepsy are lacking. However, there are a handful of sporadic research studies exploring the epidemiological dynamics of childhood epilepsy [5-7]. For instance, a study by Alonazi et al. (2018) reported that 44% of the children had epilepsy of unknown origin, while 43% were diagnosed with structural or metabolic epilepsy [5].

In a review study, it has been reported that pediatric epilepsy patients may exhibit, to a certain extent, cognitive impairment; this association has been confirmed in the literature. In fact, slowed psychomotor response and poor memory performance have also been observed in childhood idiopathic epilepsy [8]. Therefore, an assessment of incidence, prevalence, deaths, years of life lost (YLLs), years lived with disability (YLDs), and DALYs related to childhood idiopathic epilepsy over time in Saudi Arabia is required to identify the overall trends and implications for intervention. The aim of the present study is to report the incidence, prevalence, deaths, YLLs, YLDs, and DALYs of childhood idiopathic epilepsy by age, gender, and year in Saudi Arabia using data from the GBD 2019 collaboration from the year 1990 to 2019 [9].

## Materials and methods

Overview of the Global Burden of Disease (GBD) 2019

The data on the burden of childhood (children aged 0-14 years) idiopathic epilepsy in Saudi Arabia was obtained from the GBD 2019. The GBD is spearheaded by the Institute of Health Metrics and Evaluation (IHME) which provides wide-ranging worldwide data on 369 diseases and injuries, 87 risk factors, and 286 etiologies of death by age and gender, in 21 geographical regions comprised of 204 countries and territories. The GBD collaboration offers a methodical and detailed global, national, and regional data on incidence, prevalence, deaths, and health loss so that healthcare measures can be planned accordingly to mitigate the burden of disease [9].

Epidemiological data estimation of childhood idiopathic epilepsy burden

An earlier study has already described in detail the estimation of the global disease burden [10]. The epidemiological data on idiopathic epilepsy for children aged 0-14 years in Saudi Arabia from 1990 to 2019 were acquired from the website of IHME GBD 2019 (https://www.healthdata.org/gbd/2019) where the data is freely accessible. “GBD Results” and the “Interact with the data visualization” tool were used to retrieve relevant data online from the IHME GBD website (https://vizhub.healthdata.org/gbd-results/). We extracted the data of annual incidence, prevalence, deaths, YLLs, YLDs, and DALYs for the last three decades i.e. from 1990 to 2019, stratified by gender.

Definition of idiopathic epilepsy

According to IHME GBD, idiopathic epilepsy is defined as recurrent and unprovoked seizures with no identifiable underlying disease and is believed to stem from genetic deviation. The case definition of idiopathic epilepsy also includes patients with active epilepsy having at least one seizure in the last five years, irrespective of treatment [11].

Standards of data reporting

In this study, incidence, prevalence, deaths, YLLs, YLDs, and DALYs cases were reported as numbers and rates (per 100,000 population). The percentage change column indicates the annual mean percentage change during the years from 1990 to 2019. The 95% UIs were also reported for all the measures. The GBD studies estimate UIs to account for ambiguity in primary sources with regard to data and modelling, errors in data, and data handling. The standardized modelling method for calculation of the incidence, prevalence, deaths, YLLs, YLDs, and DALYs cases, rates (per 100,000 population), and gender-wise epidemiological patterns has been described previously [10, 12, 13]. Figures for the study were developed through GraphPad Prism v. 9 software (GraphPad Prism, Inc., La Jolla, CA). All the statistical analyses and estimations were conducted primarily by the IHME GBD Results Tool software [14].

## Results

Incidence of childhood idiopathic epilepsy

**Figure 1:**
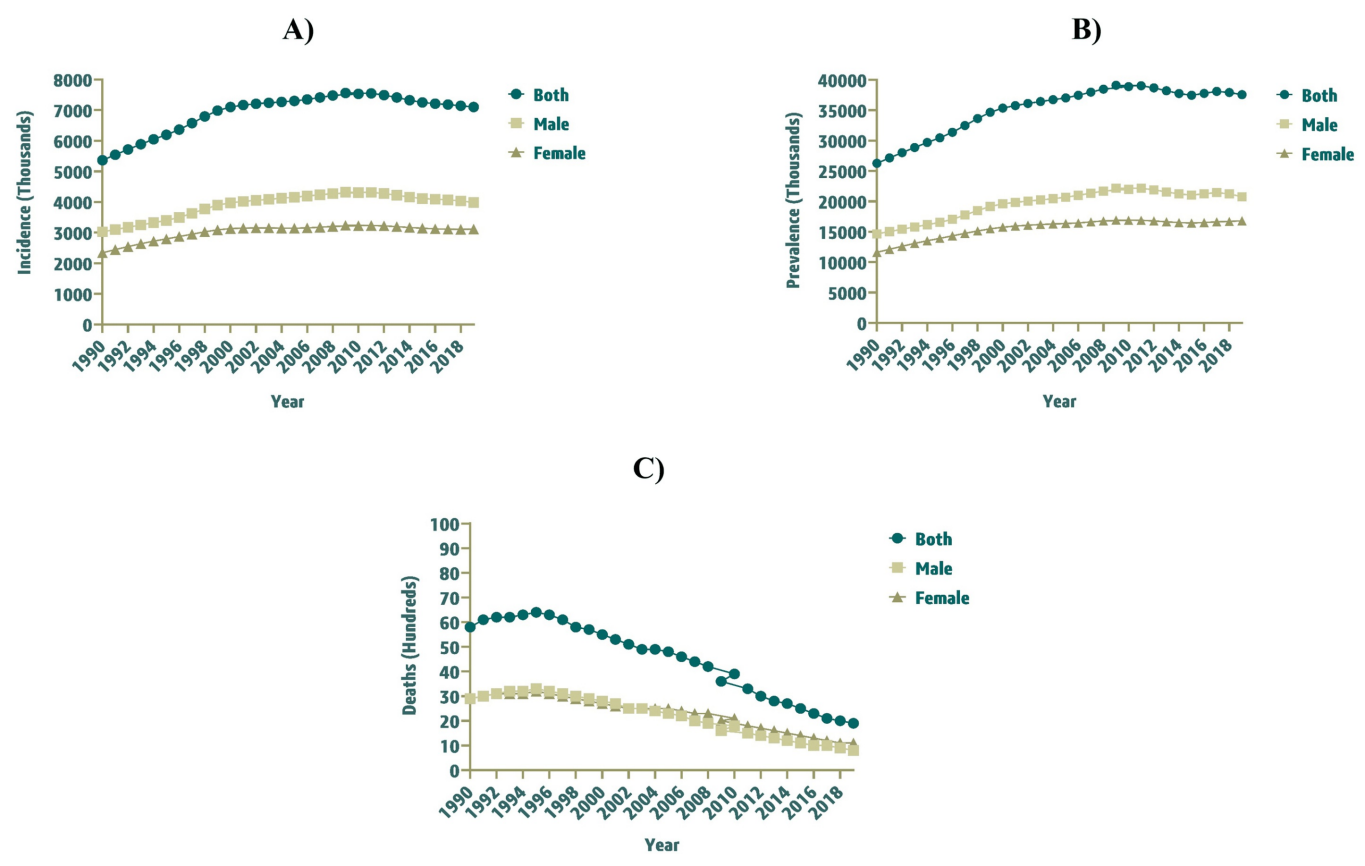
Incidence, Prevalence, and Deaths of Idiopathic Epilepsy Among Children (0–14 Years) in Saudi Arabia From 1990–2019, Stratified by Gender. A) Incidence, B) Prevalence, and C) Deaths.

In 1990, the incident cases of childhood idiopathic epilepsy were 5,365 (95% UI: 1,312 to 10,572) in Saudi Arabia, which increased to 7,101 (95% UI: 2,011 to 13,113) by 2019. The incidence rate (per 100,000 population) was 80.42 (95% UI: 19.67 to 158.45) and 100.78 (95% UI: 28.54 to 186.10) in 1990 and 2019, respectively. Overall, the incidence rate (per 100,000 population) was higher for males i.e. 88.92 (95% UI: 21.78 to 172.67) in 1990 and 107.76 (95% UI: 29.00 to 199.80) in 2019. The incidence of childhood idiopathic epilepsy in Saudi Arabia increased by 32.36% (95% UI: -61.98 to 405.94) from 1990 to 2019 (Table 1). Figure 1 demonstrates the trend of incidence, prevalence, and deaths of childhood idiopathic epilepsy among children (0-14 years) in Saudi Arabia from 1990 to 2019.

**Table 1:** Incidence, Prevalence, and Deaths in Numbers and Rates (per 100,000) of Childhood Idiopathic Epilepsy in Saudi Arabia by Gender (1990 and 2019), With Percentage Change Between 1990 and 2019 Numbers in parentheses represent 95% uncertainty intervals.

Parameters	Incidence		Prevalence		Deaths	
	Numbers	Rates (per 100,000)	Numbers	Rates (per 100,000)	Numbers	Rates (per 100,000)
1990						
Both Genders	5,365 (1,312 to 10,572)	80.42 (19.67 to 158.45)	26,275 (6,678 to 48,586)	393.82 (100.10 to 728.24)	58 (33 to 104)	0.87 (0.50 to 1.56)
Males	3,026 (741 to 5,875)	88.92 (21.78 to 172.67)	14,660 (3,779 to 27,249)	430.85 (111.06 to 800.85)	29 (16 to 48)	0.86 (0.48 to 1.42)
Females	2,340 (578 to 4,625)	71.57 (17.67 to 141.47)	11,615 (2,944 to 21,687)	355.29 (90.07 to 663.37)	29 (15 to 63)	0.89 (0.45 to 1.91)
2019						
Both Genders	7,101 (2,011 to 13,113)	100.78 (28.54 to 186.10)	37,575 (10,571 to 65,307)	533.24 (150.02 to 926.80)	19 (13 to 33)	0.27 (0.18 to 0.46)
Males	3,987 (1,073 to 7,391)	107.76 (29.00 to 199.80)	20,767 (5,773 to 36,262)	561.37 (156.06 to 980.25)	8 (5 to 14)	0.22 (0.14 to 0.39)
Females	3,115 (893 to 5,652)	93.06 (26.69 to 168.85)	16,808 (4,666 to 29,239)	502.16 (139.41 to 873.56)	11 (6 to 21)	0.32 (0.19 to 0.64)
Percentage Change (1990-2019)						
Both Genders	32.36 (-61.98 to 405.94)	25.32 (-64.00 to 379.03)	43.01 (-59.20 to 449.19)	35.40 (-61.37 to 419.98)	-67.48 (-81.56 to -31.20)	-69.21 (-82.54 to -34.86)
Males	31.76 (-61.98 to 420.61)	21.19 (-65.03 to 378.84)	41.66 (-59.05 to 445.43)	30.29 (-62.34 to 401.67)	-71.58 (-83.93 to -32.14)	-73.86 (-85.22 to -37.58)
Females	33.13 (-61.78 to 418.04)	30.03 (-62.67 to 405.97)	44.71 (-58.92 to 465.40)	41.34 (-59.87 to 452.22)	-63.36 (-83.22 to -14.95)	-64.21 (-83.61 to -16.94)

Prevalence of childhood idiopathic epilepsy

The count of prevalent cases of childhood idiopathic epilepsy in Saudi Arabia was 26,275 (95% UI: 6,678 to 48,586) in 1990, which increased to 37,575 (95% UI: 10,571 to 65,307) by 2019. Therefore, the prevalence rate (per 100,000 population) also witnessed an increase from 393.82 (95% UI: 100.10 to 728.24) in 1990 to 533.24 (95% UI: 150.02 to 926.80) in 2019. The overall prevalence rate (per 100,000 population) was found to be greater for the male gender with 430.85 (95% UI: 111.06 to 800.85) in 1990 and 561.37 (95% UI: 156.06 to 980.25) in 2019. The percentage change, between 1990 and 2019, noted for prevalent cases of childhood idiopathic epilepsy was 43.01% (95% UI: -59.20 to 449.19) (Table 1).

Death burden of childhood idiopathic epilepsy

The number of mortalities because of childhood idiopathic epilepsy in Saudi Arabia was 58 (95% UI: 33 to 104) in 1990 and decreased to 19 (95% UI: 13 to 33) in 2019. The death rate (per 100,000 population) was 0.87 (95% UI: 0.50 to 1.56) and 0.27 (95%UI: 0.18 to 0.46) in 1990 and 2019, respectively. For the female gender, the death rate (per 100,000 population) was higher i.e. 0.89 (95% UI: 0.45 to 1.91) in 1990, which substantially decreased to 0.32 (95% UI: 0.19 to 0.64) in 2019. The number of deaths because of childhood idiopathic epilepsy in Saudi Arabia declined by -67.48% (95% UI: -81.56 to -31.20) from 1990 to 2019 (Table 1).

Years of life lost - childhood idiopathic epilepsy

**Figure 2:**
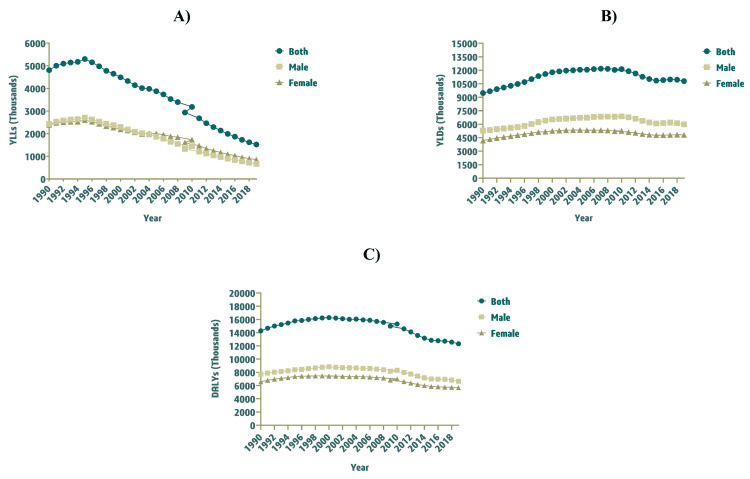
YLLs, YLDs, and DALYs of Idiopathic Epilepsy Among Children (0–14 Years) in Saudi Arabia from 1990–2019, Stratified by Gender. A) YLLs, B) YLDs, and C) DALYs. Abbreviations: YLLs, years of life lost; YLDs, years lived with disability; DALYs, disability-adjusted life years

The YLLs of childhood idiopathic epilepsy in Saudi Arabia were 4,808 (95% UI: 2,700 to 8,652) in 1990, which substantially declined to 1,525 (95% UI: 1,017 to 2,702) in 2019. The YLLs rate (per 100,000 population) was 72.06 (95% UI: 40.47 to 129.69) and 21.64 (95% UI: 14.43 to 38.35) in 1990 and 2019, respectively. While the YLLs rate (per 100,000 population) was almost similar for both genders in 1990, it was much higher for females in 2019 i.e. 25.51 (95% UI: 15.29 to 52.76). Between 1990 and 2019, the YLLs decreased by -68.29% (95% UI: -82.18 to -32.21) (Table 2). Figure 2 demonstrates the trend of YLLs, YLDs, and DALYs of childhood idiopathic epilepsy among children (0-14 years) in Saudi Arabia from 1990 to 2019.

**Table 2:** YLLs, YLDs, and DALYs in Numbers and Rates (per 100,000) of Childhood Idiopathic Epilepsy in Saudi Arabia by Gender (1990 and 2019), With Percentage Change Between 1990 and 2019 Abbreviations: YLLs, years of life lost; YLDs, years lived with disability; DALYs, disability-adjusted life years Numbers in parentheses represent 95% uncertainty intervals.

Parameters	YLLs		YLDs		DALYs	
	Numbers	Rates (per 100,000)	Numbers	Rates (per 100,000)	Numbers	Rates (per 100,000)
1990						
Both Gender	4,808 (2,700 to 8,652)	72.06 (40.47 to 129.69)	9,466 (2,170 to 21,251)	141.88 (32.53 to 318.52)	14,273 (6,242 to 26,114)	213.94 (93.57 to 391.42)
Males	2,426 (1,332 to 4,044)	71.30 (39.14 to 118.85)	5,283 (1,167 to 12,120)	155.26 (34.29 to 356.20)	7,709 (3,360 to 14,401)	226.56 (98.75 to 423.24)
Females	2,382 (1,191 to 5,243)	72.85 (36.42 to 160.37)	4,183 (929 to 9,476)	127.95 (28.42 to 289.86)	6,565 (2,820 to 11,999)	200.80 (86.26 to 367.04)
2019						
Both Gender	1,525 (1,017 to 2,702)	21.64 (14.43 to 38.35)	10,796 (2,586 to 24,378)	153.22 (36.70 to 345.96)	12,321 (3,988 to 25,617)	174.85 (56.60 to 363.54)
Males	671 (411 to 1,194)	18.13 (11.10 to 32.29)	5,977 (1,504 to 13,348)	161.58 (40.65 to 360.81)	6,648 (2,165 to 14,349)	179.71 (58.52 to 387.89)
Females	854 (512 to 1,766)	25.51 (15.29 to 52.76)	4,819 (1,078 to 10,777)	143.97 (32.21 to 321.98)	5,673 (1,848 to 11,663)	169.48 (55.20 to 348.45)
Percentage Change (1990-2019)						
Both Gender	-68.29 (-82.18 to -32.21)	-69.97 (-83.13 to -35.81)	14.06 (-73.38 to 398.26)	7.99 (-74.80 to 371.75)	-13.68 (-71.64 to 132.22)	-18.27 (-73.15 to 119.87)
Males	-72.35 (-84.46 to -33.40)	-74.57 (-85.71 to -38.75)	13.15 (-73.65 to 375.97)	4.07 (-75.76 to 337.79)	-13.76 (-73.93 to 137.13)	-20.68 (-76.02 to 118.11)
Females	-64.15 (-83.72 to -15.66)	-64.99 (-84.10 to -17.63)	15.21 (-73.19 to 397.99)	12.52 (-73.81 to 386.38)	-13.58 (-71.83 to 142.34)	-15.60 (-72.49 to 136.69)

Years lived with disability - childhood idiopathic epilepsy

In 1990, the YLDs of childhood idiopathic epilepsy were 9,466 (95% UI: 2,170 to 21,251) in Saudi Arabia, which slightly increased to 10,796 (95% UI: 2,586 to 24,378) by 2019. The YLDs rate (per 100,000 population) was 141.88 (95% UI: 32.53 to 318.52) in 1990 and 153.22 (95% UI: 36.70 to 345.96) in 2019. Overall, the YLDs rate (per 100,000 population) was higher for males i.e. 155.26 (95% UI: 34.29 to 356.20) in 1990 and 161.58 (955 UI: 40.65 to 360.81) in 2019. The YLDs of childhood idiopathic epilepsy in Saudi Arabia observed an increase of 7.99% (95% UI: -74.80 to 371.75) between 1990 and 2019 (Table 2).

Disability-adjusted life years - childhood idiopathic epilepsy

The DALYs of childhood idiopathic epilepsy in Saudi Arabia were 14,273 (95% UI: 6,242 to 26,114) in 1990 and 12,321 (95% UI: 3,988 to 25,617) in 2019. The DALYs rate (per 100,000 population) was 213.94 (95% UI: 93.57 to 391.42) and 174.85 (95% UI: 56.60 to 363.54) in 1990 and 2019, respectively. For males, the DALYs rate (per 100,000 population) was higher with 226.56 (95% UI: 98.75 to 423.24) in 1990, which dropped to 179.71 (95% UI: 58.52 to 387.89) in 2019. From 1990 to 2019, the DALYs decreased by -13.68% (95% UI: -71.64 to 132.22) (Table 2).

## Discussion

Epilepsy is among the most frequent, chronic, non-communicable neurological disorders in children. The documented incidence of epilepsy is diverse between developed and developing countries. In industrialized nations, the annual incidence is estimated to be 33.3 to 82 per 100,000 cases, while the annual incidence of 187 per 100,000 cases has been reported for developing nations [15, 16]. It is cited that about one-third of the epilepsy cases emerge due to acquired brain insults such as injuries during childbirth or brain tumors, whereas remaining epilepsy cases are thought to be caused by genetic predispositions such as monogenic and polygenic inheritance [17]. A large number of epilepsy diagnoses in children <15 years stem from unknown origin, i.e. idiopathic [18]. There is no concise evidence regarding the epidemiology of childhood idiopathic epilepsy in Saudi Arabia. This is the first study to review and report GBD 2019 data regarding the burden of childhood idiopathic epilepsy among children aged 0-14 years in the last three decades in the Kingdom of Saudi Arabia.

Epidemiological studies such as GBD help in the identification of disease trends and associated etiological factors [14]. According to our assessment of the GBD 2019 collaborators' study data, between 1990 and 2019, the incidence, prevalence, and YLDs of idiopathic epilepsy among children aged 0-14 years in Saudi Arabia relatively increased. The possible increase in number could be due to the wide availability of electroencephalogram testing. The highest incidence, prevalence, and YLD numbers and rates of childhood idiopathic epilepsy were observed in females. Regarding the epidemiological distribution of childhood idiopathic epilepsy, only sporadic studies have been performed.

Between 1990 and 2019, a decline was witnessed in the number of deaths of children with idiopathic epilepsy in Saudi Arabia. Similarly, a reduction in DALY rates, a thorough measure of the disease burden, was also noted. This finding perhaps highlights the treatment advancements and improved access to treatment leading to a reduced risk of mortality and disease severity. Earlier research studies with large retrospective and prospective cohorts suggest that the prognosis is favorable for children with idiopathic epilepsy i.e. the remission rate was almost 100% for children with benign epilepsy with centrotemporal spikes by mid-adolescence [19-23].

It is important to note that factors like socioeconomic status have been assumed to have a bi-directional relationship with epilepsy. This bi-directional relationship has also been found to exist with other neurological disorders [24]. Besides low-middle-income countries, individuals from socioeconomically underprivileged backgrounds in high-income countries have been shown to be vulnerable to epilepsy [25-27].

There are a few limitations of the study that need to be discussed. Firstly, this was the secondary analysis of the online GBD data i.e. data collection, collation, and statistical analyses for various measures such as incidence, prevalence, deaths, YLLs, YLDs, and DALYs were performed exclusively by GBD collaborators. Secondly, the accuracy and reliability of the GBD estimate rely on the quality of the data used by IHME, which is primarily obtained from administrative registers, disease registers, surveillance statistics, and population censuses for modeling and estimation of relevant indicators. Most importantly, we do not know the regional distribution of childhood idiopathic epilepsy in Saudi Arabia as regional epidemiology cannot be determined through GBD results. Also, we do not know how the socioeconomic status of a family would have affected the epidemiologic measures and outcomes of childhood idiopathic epilepsy in Saudi Arabia.

Primary research studies are indeed required from Saudi Arabia to understand the regional differences, if any, of childhood idiopathic epilepsy in Saudi Arabia, including gender-related and socioeconomic differences in its epidemiology. Moreover, it is equally important to assess the impact of childhood idiopathic epilepsy on the psycho-cognitive development and quality of life of children. We also believe that further research studies in this regard would direct the stakeholders to mobilize healthcare resources for this particular population. 

## Conclusions

In conclusion, the present study provides an assessment of the epidemiology of childhood idiopathic epilepsy burden and its three-decade trend in the Kingdom of Saudi Arabia from GBD data. In the last 30 years, the incidence and prevalence of childhood idiopathic epilepsy have modestly increased in Saudi Arabia from 1990 to 2019, but the number of deaths has fallen substantially. Notwithstanding declining deaths, an increase in YLDs due to idiopathic epilepsy in children from 1990 to 2019 was noted. Further research studies are needed from Saudi Arabia to understand the regional, gender-based, and socioeconomic differences of childhood idiopathic epilepsy and its impact on psycho-cognitive development and quality of life of children.
